# Oscillation, Conduction Delays, and Learning Cooperate to Establish Neural Competition in Recurrent Networks

**DOI:** 10.1371/journal.pone.0146044

**Published:** 2016-02-03

**Authors:** Hideyuki Kato, Tohru Ikeguchi

**Affiliations:** 1 School of Engineering, Tokyo University of Technology, Tokyo Japan; 2 Faculty of Engineering Division I, Tokyo University of Science, Tokyo, Japan; McGill University, CANADA

## Abstract

Specific memory might be stored in a subnetwork consisting of a small population of neurons. To select neurons involved in memory formation, neural competition might be essential. In this paper, we show that excitable neurons are competitive and organize into two assemblies in a recurrent network with spike timing-dependent synaptic plasticity (STDP) and axonal conduction delays. Neural competition is established by the cooperation of spontaneously induced neural oscillation, axonal conduction delays, and STDP. We also suggest that the competition mechanism in this paper is one of the basic functions required to organize memory-storing subnetworks into fine-scale cortical networks.

## Introduction

In mice experiments [1, 2], a memory is recalled when neurons that are active during a learning process are activated with optogenetic stimulation. A specific memory is considered to be stored in a subnetwork consisting of a small population of neurons. For such memory formation, competition among neurons might be necessary to embed memory-storing subnetworks into neural circuits [3–6]. Furthermore, synaptic plasticity is thought to play a critical role in subnetwork organization [7, 8].

In the last decade, studies in the field of neuroscience have revealed that synaptic modification depends on presynaptic and postsynaptic neuronal activities; this is called spike timing-dependent synaptic plasticity (STDP) [9–12]. Experimental observations suggest that the precise timing of presynaptic and postsynaptic neuronal action potentials is a crucial factor in information processing and/or memory formation in the brain. Therefore, STDP is thought to be one of the mechanisms to encode information into patterns of their synaptic weights [13, 14].

Based on experimental observations, several studies have proposed models of STDP window functions [15–18]. For example, Song et al. [15] modeled synaptic learning that is independent of the synaptic weight; this is called an additive model or a hard-bound model. In contrast, Rossum et al. Rossum et al. [16] and Rubin et al. [17] proposed a model that linearly depends on the synaptic weight; this is called a multiplicative model or a soft-bound model. The dependence on synaptic weights in STDP models is generalized [17, 18]. Temporal development and equilibrium states of synaptic distribution have been well investigated through these STDP models and are supported by the Fokker-Plank theorem [16–18]. Extended models were also recently proposed [19, 20].

Several studies on memory formation have reported that STDP has the ability to establish specific structures like neural clusters in recurrent neural networks [21–28]. This clustering can be regarded as a form of neural competition. In a spontaneously organized network, neurons in a cluster simultaneously emit spikes that force neurons in the next cluster to fire. Therefore, the synchrony of each cluster is successively conducted to the next cluster and is evoked cyclically in the network. The reason for the synchrony distributed in time is that individual neuronal activities are under the theoretical limit of integration or the limit cycle. These conditions are, however, unusual in the cortical and hippocampal neurons in the homeostatic process [29, 30]. In addition, it has been presented that STDP enables recurrent neural networks to organize a feed-forward topology in presence of background noise, that does not lead neurons to the theoretical limit of integration or the limit cycle [23].

In this paper, we aimed to present that STDP leads excitable neurons in a sparse recurrent network to competition that might be essential in memory formation. Detailed analyses of neural mechanisms showed that competition is accomplished by the cooperation of spontaneously induced neural oscillations, axonal conduction delays, and STDP.

## Materials and Methods

### Neural network model

We employ Izhikevich’s simple neuron model as the basis of our neural network [31, 32]. This model is not only computationally effective as the leaky integrate-and-fire model, but can also realize firing patterns as rich as those in the Hodgkin-Huxley model [33]. Dynamics of the *j*th (*j* = 1, 2, …, *N*) neuron is described by the following two-dimensional ordinary differential equations:

$$\begin{array}{ccc} {\dot{v}}_{j} & = & 0.04v_{j}^{2}+5v_{j}+140-u_{j}+I_{j}\left(t\right), \end{array}$$ (1)

$$\begin{array}{ccc} {\dot{u}}_{j} & = & a_{j}\left(b_{j}v_{j}-u_{j}\right), \end{array}$$ (2)

where *v*_*j*_ is the membrane potential and *u*_*j*_ is the recovery variable of the *j*th neuron. The membrane potential and the recovery variable of the neuron model is reset to *c*_*j*_ mV and *u*_*j*_ + *d*_*j*_ when *v*_*j*_ reached 30*mV*. The variable *I*_*j*_(*t*) represents inputs to the *j*th neuron at time *t*. The inputs are the summation of external inputs ($I_{j}^{\text{ext}}\left(t\right)$) and synaptic inputs ($I_{j}^{\text{syn}}\left(t\right)$). For the sake of simplicity, we model the synaptic inputs with the delta function *δ*(⋅),

$$\begin{array}{c} I_{j}^{\text{syn}}\left(t\right)=\sum_{i=1}^{N}\sum_{k=1}^{n_{j,i}}w_{ij}\delta \left(t-t_{i,k}\right), \end{array}$$ (3)

where *w*_*ij*_ is the synaptic weight from the *i*th neuron to the *j*th neuron, and *t*_*i*, *k*_ is the arrival time of the *k*th (*k* = 1, 2, …, *n*_*j*, *i*_ where *n*_*j*, *i*_ represents the number of spikes of the *i*th presynaptic terminal of the *j*th neuron) spike at the *i*th presynaptic terminal. Our neural network consists of *N* (= 1,000) neurons including both excitatory and inhibitory neurons. The ratio of the excitatory neurons to the inhibitory neurons is 4: 1 [34]. In this paper, we use regular-spiking excitatory neurons and fast-spiking inhibitory neurons. The parameters for the excitatory neurons are set as *a*_*j*_ = 0.02, *b*_*j*_ = 0.2, *c*_*j*_ = − 65, and *d*_*j*_ = 8 and those for the inhibitory neurons are set as *a*_*j*_ = 0.1, *b*_*j*_ = 0.2, *c*_*j*_ = − 65, and *d*_*j*_ = 2 [31, 32]. These neurons are randomly connected. Because, in many experimental studies, the connection probability has been estimated between 0.1 and 0.3 [35–39], we choose 0.1 connection probability. In the neural network, no connections exist between any pairs of the inhibitory neurons. Furthermore, no neurons are self-connected. Excitatory connections have conduction delays of 1 to 10 ms, with a uniform distribution [40]. A time of 1 ms is required to transmit spikes on all inhibitory connections [40]. It has been shown that dendritic delays tend to strengthen self-feedback, whereas axonal delays weaken it [41, 42]. We assume that the conduction delays are only axonal.

Each neuron in the network receives an independent and uncorrelated Poisson spike train with the fixed firing rate of *f* spk/s during our simulation through a non-plastic excitatory feed-forward connection. The spike train is statistically identical for both excitatory and inhibitory neurons. In the simulations, we test *f* = 1, 10, and 40 spk/s. The reason for the usage of the Poisson spike train is based on the observation that *in vivo* neuronal behaviors in cortical areas are highly irregular [29]. The amplitude of each spike in the spike sequence is set to 20 mV. In other words, $I_{j}^{\text{ext}}\left(t\right)=20$ in Eq (1), which corresponds to a suprathreshold input when a neuron is in the resting state. A spike train for a neuron is statistically equivalent to spike trains for the other neurons.

All excitatory synaptic weights are initially set to 6 mV, except for the simulation in the section of Independence of initial distribution of plastic synapses on neural competition. whereas all inhibitory synaptic weights are set to − 5 mV. Research has shown the significance of synaptic types [12], and therefore STDP is applied only to the excitatory synapses between excitatory neurons in the network.

STDP is a type of Hebbian synaptic plasticity that has attracted considerable attention [9, 11, 12]. In this synaptic plasticity, if a postsynaptic action potential follows a presynaptic action potential within tens of milliseconds, the synaptic weight between them is strengthened; this is long-term potentiation (LTP). On the other hand, a synapse is depressed if a presynaptic action potential follows a postsynaptic action potential, which is long-term depression (LTD). In this paper, we adopt the additive STDP rule proposed by Song et al. [15]. Its window function is expressed in terms of the exponential functions as follows:

$$\Delta w_{ij}=\;\left\{ \begin{array}{l} \;\lambda e^{-\left|\Delta t\right|/\tau }\text{ i}f\;\Delta t>0,\\ -\alpha \lambda e^{-\left|\Delta t\right|/\tau }\text{ o}therwise, \end{array}\right.$$ (4)

where Δt = *t*_*j*_ − *t*_*i*_ is the relative spike timing between a presynaptic terminal and a postsynaptic neuron. Hard bounds is assumed for plastic synapses. Therefore, the plastic synapses are constrained in the range of [0, *w*_*max*_], where *w*_*max*_ is set to 10 (except for the simulation in the section of Influence of neural network parameters on neural competition). The variable *λ*(= 0.1 mV) is the learning rate [40]. The variable *α* is the degree of asymmetry between LTD and LTP. This parameter is typically set to 1.2, but is varied in the simulation in the section of Influence of neural network parameters on neural competition. We use the same time constant *τ* (= 20 ms) for both the LTP and the LTD [12]. Synaptic derivatives are changed at individual firing events, and actual synaptic weights are updated once a second. In all numerical simulations, spike interactions in the STDP rule are limited to nearest-neighbor pairs, except for the simulation in the section of Influence of spike interactions in STDP on neural competition. [42]. Potentiation and depression, which are independent of firing events, are also included in the synaptic modifications as in Izhikevich [40].

### Strength correlations

To quantify network structures, the degree of individual nodes is usually measured. If connections in networks are directional, we can take into account two types of degrees: indegree and outdegree. The indegree and the outdegree of the *j*th node can be defined by the total numbers of incoming (afferent) and outgoing (efferent) connections, respectively, and are expressed as

$$\begin{array}{ccc} k_{j}^{\text{in}} & = & \sum_{i=1}^{N}H_{0}\left(w_{ij}\right), \end{array}$$ (5)

$$\begin{array}{ccc} k_{j}^{\text{out}} & = & \sum_{i=1}^{N}H_{0}\left(w_{ji}\right), \end{array}$$ (6)

where *H*_0_(*x*) is the Heaviside step function in which *H*_0_(*x*) = 1 if *x* > 0; *H*_0_(*x*) = 0 if *x* ≤ 0.

A high indegree implies that a neuron is affected by many other neurons, whereas a high outdegree implies that a neuron affects many other neurons through synaptic connections. If indegrees and outdegrees of neurons are biased, the bias is visualized in a joint degree distribution matrix (JDDM). The imbalance of indegrees and/or outdegrees of neurons in a network appears in distances from the main diagonal of the matrix. Degree distributions in real networks often have the scale-invariant or scale-free property [43].

In the case of neural networks, synaptic connections do not have only directions, but also weights. The quantification of such weighted directional networks needs a natural extension of the degrees defined in Eqs (5) and (6) [44]. These are called instrength and outstrength. The instrength and the outstrength of the *j*th neuron are defined by the sum of the normalized synaptic weights of afferent and efferent connections, respectively:

$$\begin{array}{ccc} s_{j}^{\text{in}} & = & \sum_{i=1}^{N}w_{ij}/w_{\text{max}}, \end{array}$$ (7)

$$\begin{array}{ccc} s_{j}^{\text{out}} & = & \sum_{i=1}^{N}w_{ji}/w_{\text{max}}. \end{array}$$ (8)

An instrength indicates how much a neuron is affected by other neurons, whereas an outstrength indicates how much a neuron influences the other neurons. The imbalance between instrengths and outstrengths of neurons in a network is visualized in a joint strength distribution matrix (JSDM).

In the following of this study, we quantify self-organized neural network structures through STDP using the instrength and the outstrength defined by Eqs (7) and (8). Only excitatory connections between excitatory neurons in our network are plastic; therefore, we focus on a subnetwork consisting of the excitatory neurons for the network structure quantification.

To evaluate connectivity among neurons in our neural network, we introduce measures that we call instrength- and outstrength-correlation coefficient. The correlations are an extension of the degree correlation [45]. The degree correlation coefficient is usually computed from the total degree of the *j*th neuron: $k_{j}=k_{j}^{\text{in}}+k_{j}^{\text{out}}$. To calculate the degree correlation coefficient, remaining degrees are used. The remaining degree is the number that is one less than the total degree. However, in our calculation, instrengths and outstrengths are directly used. We define the instrength- and the outstrength-correlation coefficient as follows:

$$\begin{array}{c} r^{a}=\frac{M^{-1}\sum_{i,j}H_{0}\left(w_{ij}\right)\left(s_{i}^{a}-\eta_{t}\right)\left(s_{j}^{a}-\eta_{h}\right)}{\sigma_{t}^{a}\sigma_{h}^{a}}, \end{array}$$ (9)

where *M* = $\sum_{i,j}$ *H*_0_(*w*_*ij*_), $\eta_{t}^{a}=M^{-1}\sum_{i,j}H_{0}\left(w_{ij}\right)s_{i}$, $\eta_{h}^{a}=M^{-1}\sum_{i,j}H_{0}\left(w_{ij}\right)s_{j}$, $\sigma_{t}^{a}=\sqrt{M^{-1}\sum_{i,j}H_{0}\left(w_{ij}\right){\left(s_{i}^{a}-\eta_{t}^{a}\right)}^{2}}$, and $\sigma_{h}^{a}=\sqrt{M^{-1}\sum_{i,j}H_{0}\left(w_{ij}\right){\left(s_{j}^{a}-\eta_{h}^{a}\right)}^{2}}$ (See also the section of Connectivity of winner and loser neurons). The subscripts *t* and *h* denote the tail and the head of a connection and the superscript *a* indicates in or out.

### Identification of winner or loser neurons

**Fig 1:**
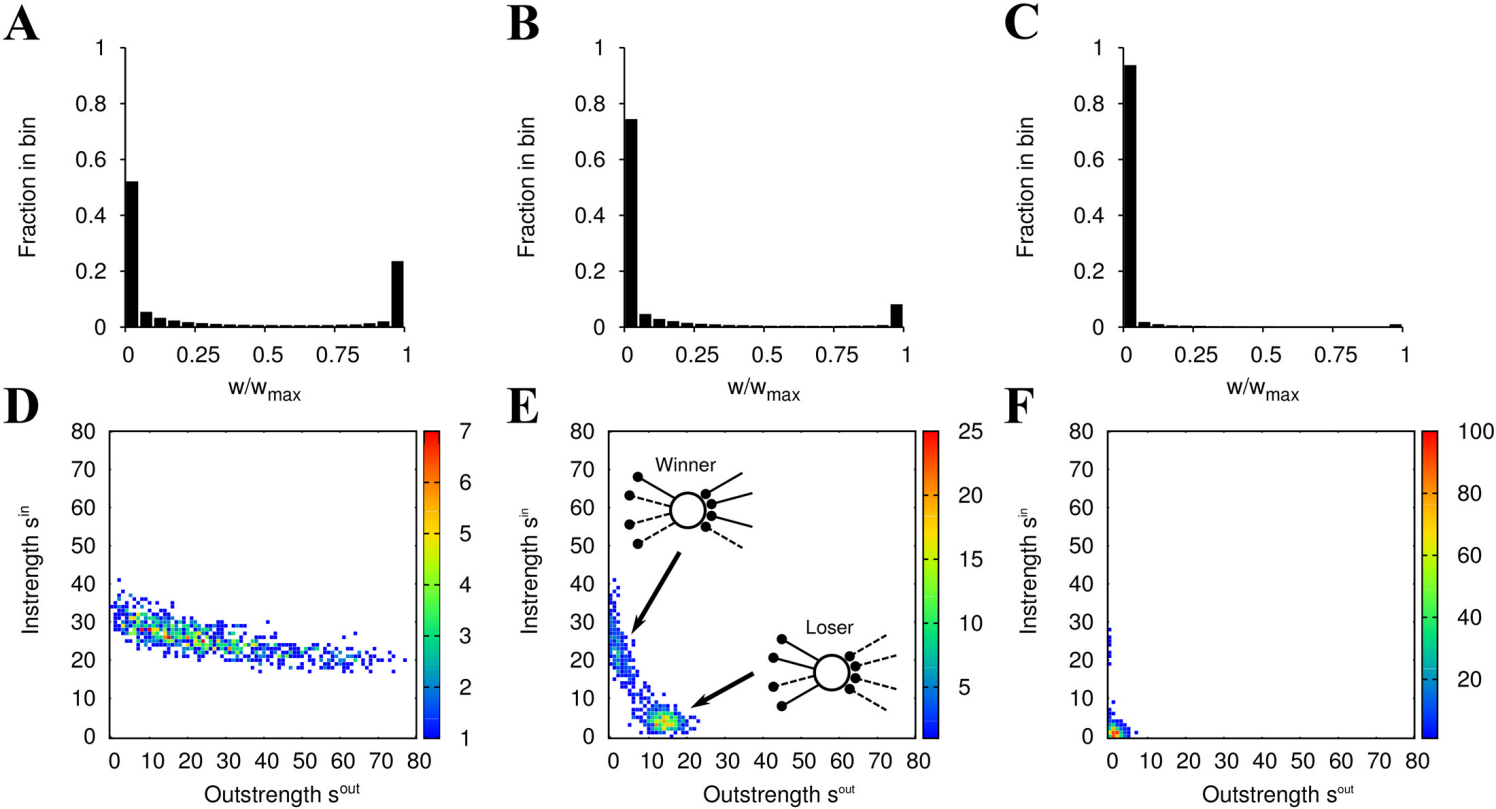
Neuronal competition in the neural network induced by STDP. (A)–(C) Histograms of plastic synaptic weights. (D)–(F) Joint strength distribution matrices (JSDMs) of excitatory neurons. The mean firing rates of the external inputs are (A), (D) 1 spk/s, (B), (E) 10 spk/s, and (C), (F) 40 spk/s. In the JSDMs, the colors represent the frequency of the excitatory neurons. In (E), the schematics of the winner and the loser neurons are illustrated. The winner neurons obtain many strong incoming connections but their many outgoing connections are weak. The loser neurons have properties opposite to those in winner neurons. All the results in this figure are generated from the networks at *t* = 3,600 s.

To identify the composition of a winner and a loser group, winner and loser neurons are defined based on their instrength. This is because, as seen in Fig 1, the instrengths tended to spread more widely than the outstrengths. As such, the threshold of the winner and the loser neurons is able to be easily determined. It should be noted that even if we have used the outstrength as a standard, our results will have been consistent in principle. The method to identify the winner groups and the loser groups is as follows. First, neurons are organized in descending order of their instrengths, and a neuron that satisfies the condition of *s*^in^ < *s*^out^ is identified. This neuron is set as the threshold and neurons before it are treated as winner neurons, and the remaining neurons are treated as loser neurons.

### Definition of phase

To characterize activities of neural networks or populations of presynaptic terminals, we also obtain phases from oscillatory firing rates. These firing rates are low-pass filtered to define the phases, and the cut-off frequency of the filter is determined to be 35 Hz based on the result of our spectral analysis in the section of Neural competition is organized in neural oscillation. After filtering, we define the phases [46] as

$$\begin{array}{c} \phi \left(t\right)=2\pi \frac{t-t_{k}}{t_{k+1}-t_{k}}-\pi , \end{array}$$ (10)

where *t*_*k*_ and *t*_*k* + 1_ correspond to any pairs of neighboring negative peaks of the oscillatory firing rate. The subtraction of *π* is to arrange the positive peak between *t*_*k*_ and *t*_*k* + 1_ at *ϕ*(*t*) = 0. We obtain the phases from the firing rates of the entire network or the presynaptic terminals.

### Estimation of firing rates

The mean firing rate of *N* neurons is estimated by the following equation:

$$\begin{array}{c} F\left(t\right)=\frac{1}{N}\sum_{j=1}^{N}f_{j}\left(t\right), \end{array}$$ (11)

where *f*_*j*_(*t*) is the firing rate of the *j*th neuron at time *t*. The firing rate of each neuron is given by

$$\begin{array}{c} f_{j}\left(t\right)=\frac{1}{T}\int_{0}^{T}\rho_{j}\left(t+\tau \right)d\tau , \end{array}$$ (12)

where *T* ( = 10 ms) is the width of a temporal window, and *ρ*_*j*_(*t*) is the spike train of the *j*th neuron:

$$\begin{array}{c} \rho_{j}\left(t\right)=\sum_{k=1}^{n_{j}}\delta \left(t-t_{j,k}\right), \end{array}$$ (13)

where *n*_*j*_ is the number of spikes of the *j*th neuron and *t*_*j*, *k*_ denotes the time of the *k*th spike of the *j*th neuron. If the neuron emits a spike *ρ*_*j*_(*t*) = 1 and otherwise *ρ*_*j*_(*t*) = 0. In the case of presynaptic terminals, we only consider the conduction delays. That is, we use the timings of the presynaptic-terminal firings instead of the timings of the somatic firings and then conduct the same estimation.

### Kendall’s correlation coefficient

Kendall’s correlation coefficient *τ*_*K*_ is a non-parametric value to quantify correlation of a paired data set. Let us define a pair of data as *x*_*i*_ and *y*_*i*_ (*i* = 1, 2, …, *m*). We describe their ranks as *X*_*i*_ and *Y*_*i*_ and consider pairs of rank data (*X*_*i*_, *Y*_*i*_). The data pairs are sorted in ascending order of *X*_*i*_. For each *Y*_*i*_ (*i* = 1, 2, …, *m* − 1), the number *P*_*i*_ satisfying *Y*_*i*_ > *Y*_*j*_ (*j* = *i* + 1, *i* + 2, …, *m*) is counted. Analogously, the number of *Y*_*i*_ < *Y*_*j*_ is described as *Q*_*i*_. Note that *P*_*i*_ + *Q*_*i*_ = *m* − *i* is always satisfied. Using the values *P*_*i*_ and *Q*_*i*_ (*i* = 1, 2, …, *m* − 1), Kendall’s correlation coefficient is computed as

$$\begin{array}{c} \tau_{K}=\frac{2}{m(m-1)}\left(\sum_{i=1}^{m-1}P_{i}-\sum_{i=1}^{m-1}Q_{i}\right), \end{array}$$ (14)

where − 1 ≤ *τ*_*K*_ ≤ 1.

### Watson’s *U*^2^-test

Watson’s *U*^2^-test is a non-parametric test for phase data. This test identifies significant differences of the mean value and/or the variance of phase distributions. Here we describe *ϕ*_*x*_(*i*) (*i* = 1, 2, …*m*_1_) and *ϕ*_*y*_(*j*) (*j* = 1, 2, …, *m*_2_) as two samples where both are sorted in ascending order. Therefore, the indices of *i* and *j* represent the ranks of phase data in *ϕ*_*x*_(*i*) and *ϕ*_*y*_(*j*), respectively. The total amount of data is *M* = *m*_1_ + *m*_2_. Next two variables *X*_*k*_ and *Y*_*k*_ (*k* = 1, 2, …, *M*) are prepared. For all the data of *ϕ*_*x*_(*i*) and *ϕ*_*y*_(*j*), the following process is repeated:

1. Set *i* = *j* = *k* = 1.
2. Compare *ϕ*_*x*_(*i*) with *ϕ*_*y*_(*j*).
3. If *ϕ*_*x*_(*i*) < *ϕ*_*y*_(*j*), *X*_*k*_ = *i*/*m*_1_ and *Y*_*k*_ = 0, otherwise *X*_*k*_ = 0 and *Y*_*k*_ = *j*/*m*_2_.
4. When *ϕ*_*x*_(*i*) < *ϕ*_*y*_(*j*), *i* is incremented, otherwise *j* is incremented.
5. *k* is incremented.
6. Repeat 2–5 until *k* = *M*.

Afterwards, *d*_*k*_ = *X*_*k*_ − *Y*_*k*_ is calculated for all *k*. Using *d*_*k*_, Watson’s *U*^2^-value is computed as

$$\begin{array}{c} U^{2}=\frac{m_{1}m_{2}}{M}\left(\frac{1}{M}\sum_{k=1}^{M}d_{k}^{2}-{\left(\frac{1}{M}\sum_{k=1}^{M}d_{k}\right)}^{2}\right). \end{array}$$ (15)

In the table of significant values, the significance of the mean value and/or the variance in two-phase distributions is evaluated.

## Results

### Equilibrium states of distributions of plastic synapses

First, we show synaptic distributions to check the temporal behavior of the synapses (Fig 1A–1C). In agreement with the previous numerical and theoretical studies with the Fokker-Plank theory [15, 17, 18, 47], it is observed in our simulations that plastic synapses in the network bimodally distribute after the long-time simulations (*t* = 3,600 s). Due to the ability of the STDP to prevent a firing rate in networks from drastically increasing, many plastic synapses go to the lower bound if the firing rate in the networks is high [15, 17, 18]. Also after *t* = 3,600 s, individual synapses continually change due to firing events. Nevertheless, the influence of these changes are small and trivial, and the form of the synaptic distributions is almost invariant (S1 Fig). Then, we regard the networks after 3,600 s as being enough stable to quantify the network organization using synaptic weights. The stability of the organized network is further discussed in the following section.

### STDP induces neural competition: emergence of winner and loser neurons

The joint strength distribution matrix (JSDM) represents the imbalance between the instrengths (*s*^in^) and the outstrengths (*s*^out^) observed in our neural network (Fig 1D–1F). The JSDM exhibits a two-dimensional Gaussian distribution before the STDP learning (results are not shown) because excitatory synapses between excitatory neurons are randomly connected and the weights of the synapses are homogeneous. After the STDP learning, the outstrengths are widely distributed, while the instrength distribution is narrow when the firing rates of the external inputs are 1 spk/s (Fig 1D).

For external inputs of 10 spk/s, the bias of the instrengths is magnified (Fig 1E). Moreover, the neurons in the neural network compete and two peaks emerge, indicating the existence of two assemblies. The neurons in one assembly have high instrength but low outstrength, while the neurons in the other assembly exhibit an opposite trend. The instrengths of the neurons in these two assemblies display clear differences. The outstrengths are also widely distributed but are narrower than those with 1 spk/s external input (Fig 1D and 1E).

In the case of external inputs with a higher firing rate of 40 spk/s, almost all excitatory neurons have similar instrengths and outstrengths, however, a few neurons achieve high instrength (Fig 1F). In comparison to the outstrengths, the instrengths form a relatively wide distribution in the space of *s*^in^–*s*^out^.

In all the cases, synaptic competition is observed, but the ratio of synapses reaching the upper-bound and the lower-bound depends on the mean firing rate of the external inputs (Fig 1A–1C). Additionally, the degree of neuronal competition changes depending on the mean firing rates (Fig 1D–1F). According to these results, the neuronal competition is related to the ratio of depressed synapses to potentiated synapses.

### Stability of neural competition through STDP

**Fig 2:**
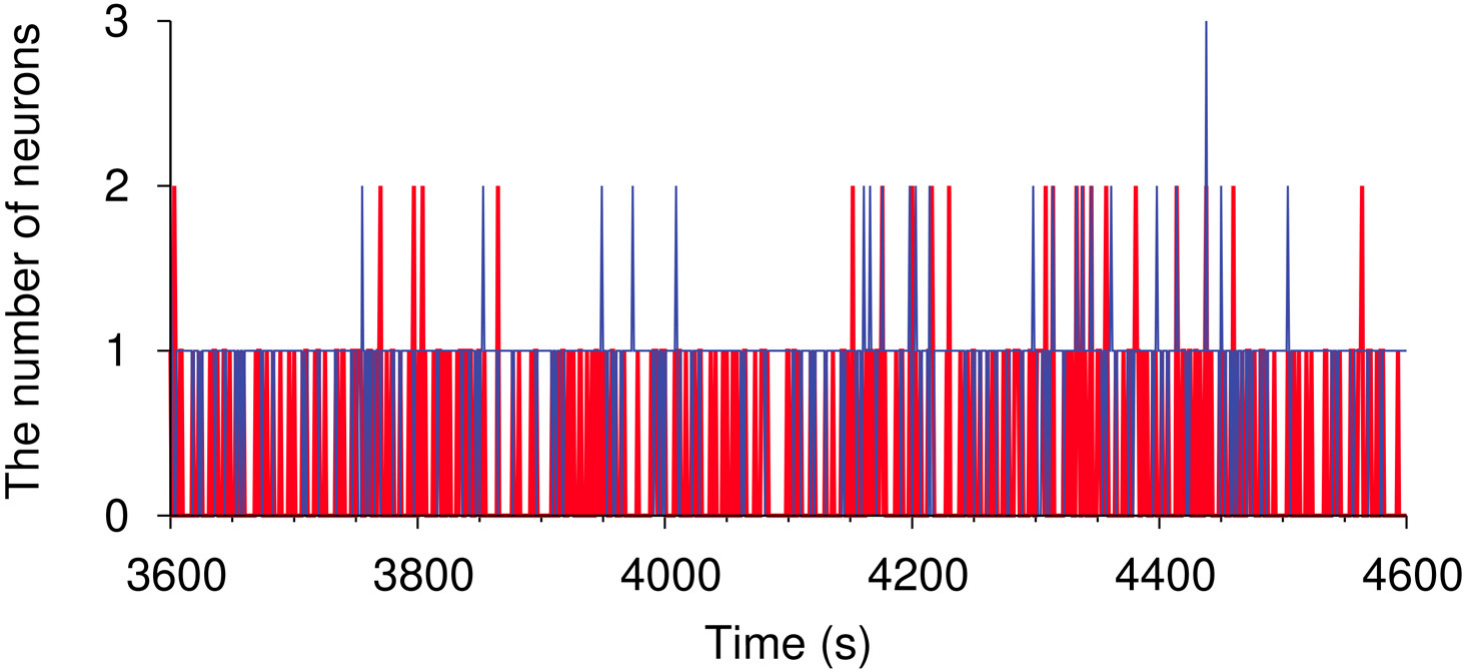
Stability of the neural competition in the STDP network. The red line indicates the number of neurons that move from the winner assembly to the loser assembly. The winner and the loser group are identified at each second. The blue line is the same as the red one, but progresses from loser to winner status.

To evaluate the stability of the neural competition, we count the number of neurons that move between the winner and the loser assembly from *t* to *t* + 1 s (Fig 2). In both cases, i.e. movement from the winner to the loser, or from the loser to the winner assemblies, a maximum of three neurons moved, corresponding to 0.38% of the total excitatory neurons. This change is negligible on a whole network. We, therefore, regard the neural competition as stable.

### Connectivity of winner and loser neurons

**Fig 3:**
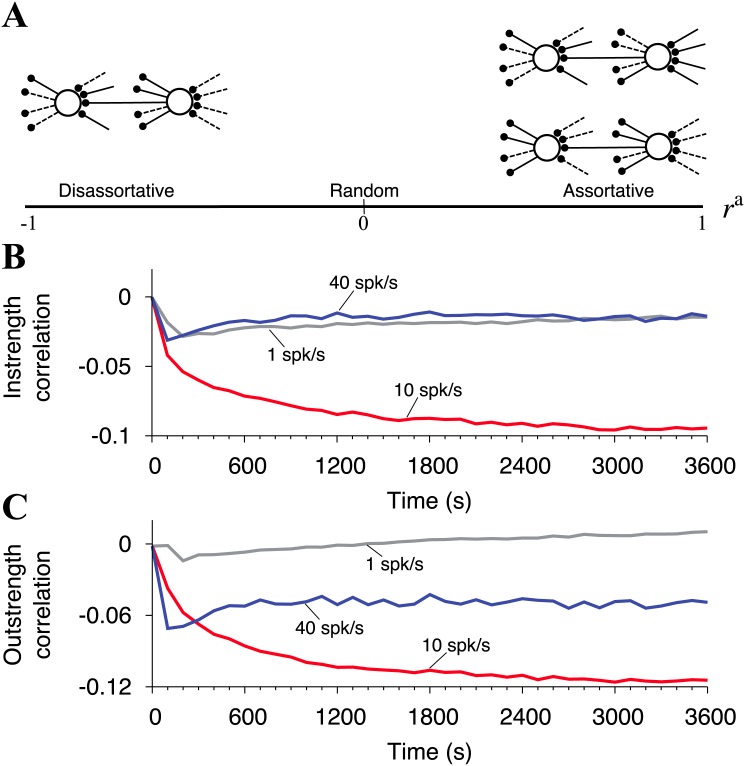
Connectivity of winner and loser neurons. (A) Schematic diagram of assortativity and disassortativity that are evaluated using the instrength- and the outstrength-correlation coefficient. Positive (negative) values of the coefficients represent assortativity (disassortativity) of networks, and zero corresponds to random networks. (B), (C) The time traces of the instrength- and the outstrength-correlation coefficient during STDP with external input firing rates of 1 spk/s (gray line), 10 spk/s (red line), and 40 spk/s (blue line). The coefficients are computed at every second.

To analyze how the competitive neurons are connected in the network, we characterize the neural network with instrength- and outstrength-correlation coefficient, *r*^in^ and *r*^out^. These coefficients quantify the similarity of neurons at the ends of connections in networks (See also Fig 3A). When two neurons at the ends of connections in a network tend to have similar instrengths or outstrengths, the coefficients are positive. When instrengths or outstrengths are dissimilar, these coefficients are negative.

The time courses of *r*^in^ and *r*^out^ are shown in Fig 3B and 3C. At *t* = 0 s, both *r*^in^ and *r*^out^ are zero in all cases because the neurons in the neural network are randomly connected through synapses under the initial condition. Evidently, *r*^in^ and *r*^out^ only significantly decrease from the initial condition at 10 spk/s. They then converge at approximately − 0.1 and − 0.12 (*P* < 0.001, *t*-test), respectively. In the other cases, *r*^in^ and *r*^out^ do not reach this level of dissimilarity. Such significant differences of the coefficient values for 10 spk/s case come from the clear competition between the winner and the loser assembly (Fig 1E). We should note that it is impossible for the coefficients to be negative at the significant level if the neurons do not compete and if the neurons in the individual assemblies have the reverse trend of the instrengths and the outstrengths as Fig 1E. Taking into account the results in Fig 1, the neurons are competitive and the dissimilar neurons tend to locate at the ends of individual connections. In other words, connections between the winner and the loser assembly are more strengthened and internal connections in the individual assemblies are easily pruned off.

In addition to the dismilarity, we also analyze the small-world property of the neural network [48]. At the beginning of the simulation (*t* = 0 s of Fig 2 in [48]), the network has as a large clustering coefficient and a characteristic path length as regular networks. The effect of the STDP leads to a much smaller characteristic path length relatively to the regular networks, although the large clustering coefficient is maintained (*t* = 600 s of Fig 2 in [48]). This indicates that the small-world network emerges in the connectivity among the winner and the loser neurons. In this study, neural competition is primary interest and the analyses therefore focus on cases of 10 spk/s-external input.

### Independence of the numbers of excitatory and inhibitory presynaptic neurons on neural competition

**Fig 4:**
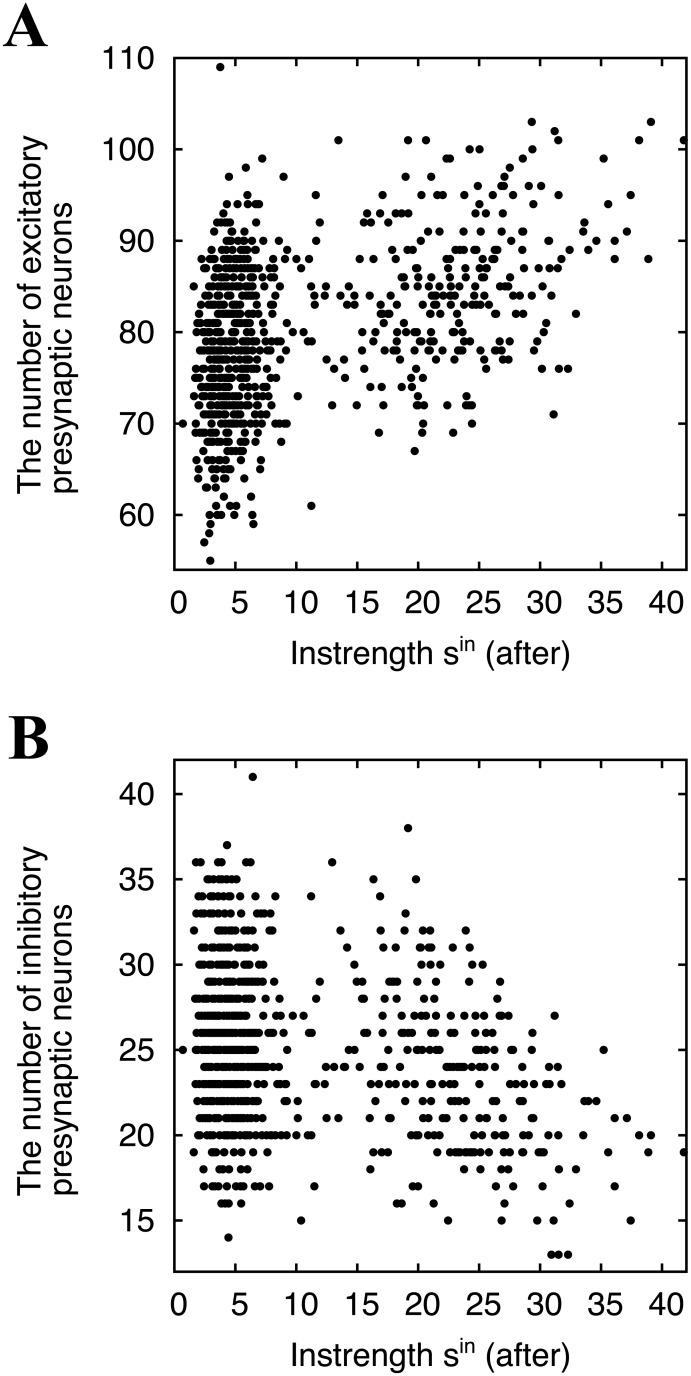
Influence of the initial conditions on the neuronal competition. Scattergram (A) shows the number of excitatory presynaptic neurons versus the instrength after learning at *t* = 3,600 s (*τ*_*K*_ = 0.18), and (B) shows the number of inhibitory presynaptic neurons versus the instrength after learning at *t* = 3,600 s (*τ*_*K*_ = − 0.3).

We have shown that neurons in the network are competitive when 10 spk/s-external inputs are given to each neuron. Since presynaptic and postsynaptic activities determine synaptic modifications, the factors determining postsynaptic activity might be the key to neural competition. When considering how to construct our network (See Materials and Methods), one of the differences of individual neurons is the number of presynaptic neurons because we have adopted statistically equivalent external inputs. Therefore, for individual neurons receiving 10 spk/s external inputs, we plot the number of excitatory presynaptic neurons against their instrengths after learning (*t* = 3,600 s) as shown in Fig 4A. They clearly do not have a linear correlation so their correlation is quantified by the Kendall correlation coefficient *τ*_*K*_—a non-parametric index (See details in Materials and Methods). However, even this coefficient cannot identify a correlation (*τ*_*K*_ = 0.18).

In the same way, the number of inhibitory presynaptic connections is plotted against the instrengths after STDP (*t* = 3,600 s) in Fig 4B. Since inhibitory synaptic weights are negative, they have a negative correlation, but the coefficient is small and negative (*τ*_*K*_ = − 0.3). Therefore, we conclude that the number of excitatory and inhibitory synapses on individual neurons has a minimal effect on whether neurons obtain many or few strong synapses.

### Independence of initial distribution of plastic synapses on neural competition

For further analysis of the independence of the initial network architecture, we also change a given distribution of excitatory synaptic weights in the initial condition and observed the JSDMs after STDP (*t* = 3,600 s) for the 10 spk/s external input samples in S1 File. The variety of the initial synaptic distribution does not affect the JSDM after learning, and STDP induces neuronal competition. The three JSDMs are in perfect agreement with Fig 1B, which indicates that the structures are robust for certain synaptic weights under the initial condition. Therefore, synaptic weights before learning has little influence on neuronal competition.

### Relation between axonal conduction delay and synaptic modifications

**Fig 5:**
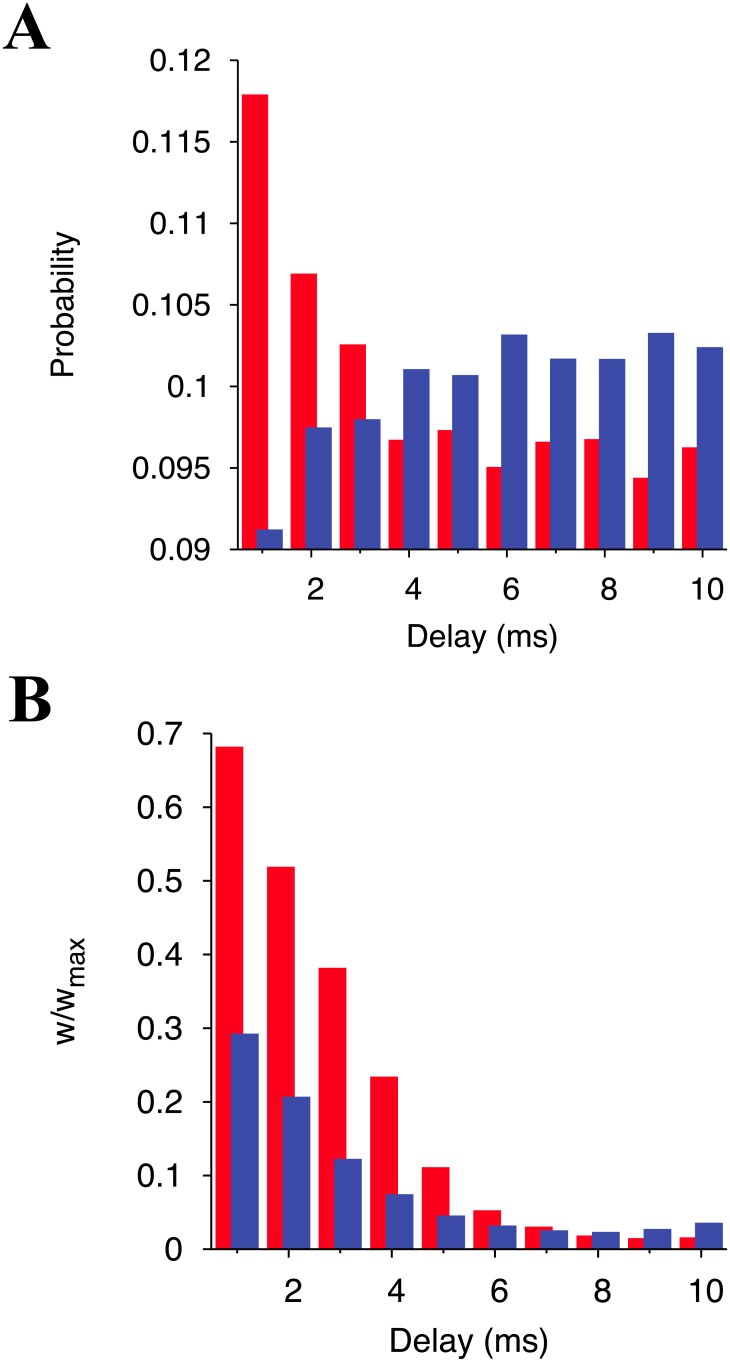
Statistics of conduction delays of synaptic connections to the winner and the loser neurons and their relation with the plastic synaptic weights. (A) The conduction delay distribution in excitatory presynaptic connections of a neuron. The value in each bin is averaged over all the winner neurons or all the loser neurons. (B) The average synaptic weight of each conduction delay. Red and blue bars represent winner and loser neurons, respectively. The data are generated from the network at *t* = 3,600 s.

Another conceivable difference in the statistics of presynaptic connections for individual neurons is the distribution of axonal conduction delays. As such, we analyze the ratio of each conduction delay to the total number of presynaptic connections for each neuron. Fig 5A shows the mean values of this ratio in the winner and the loser assembly. The smaller the conduction delays are, the higher their ratio is in the winner neurons. In contrast, the ratio of larger conduction delays is higher in the loser neurons. From this result, the high ratio of shorter-delay connections seems to be advantageous for neurons to become winners.

We also analyze the relation between the conduction delays and the mean synaptic weights after learning (*t* = 3,600 s) of both winner and loser neurons (Fig 5B). For both winner and loser neurons, the smallest delay has the largest mean weight, and the mean weight decreases as the conduction delay increases. This result indicates that smaller conduction delays lead to stronger synapses. However, during the first 5 ms of the conduction delays, the mean synaptic weights of the winner neurons are twice as strong as those of the loser neurons. This explains why winner neurons can get a high instrength but loser neurons cannot.

### Neural competition is organized in neural oscillation

We have shown that winner neurons have a higher ratio of presynaptic connections with smaller conduction delays that are capable of more potentiation. However, it is still unclear why the small delay connections of the winner neurons are more strengthened than those of the loser neurons. To unveil origins of strong potentiation of the small delay synaptic connections of the winner neurons, we observe the network activity in the STDP network because it has a strong effect on synaptic modifications.

**Fig 6:**
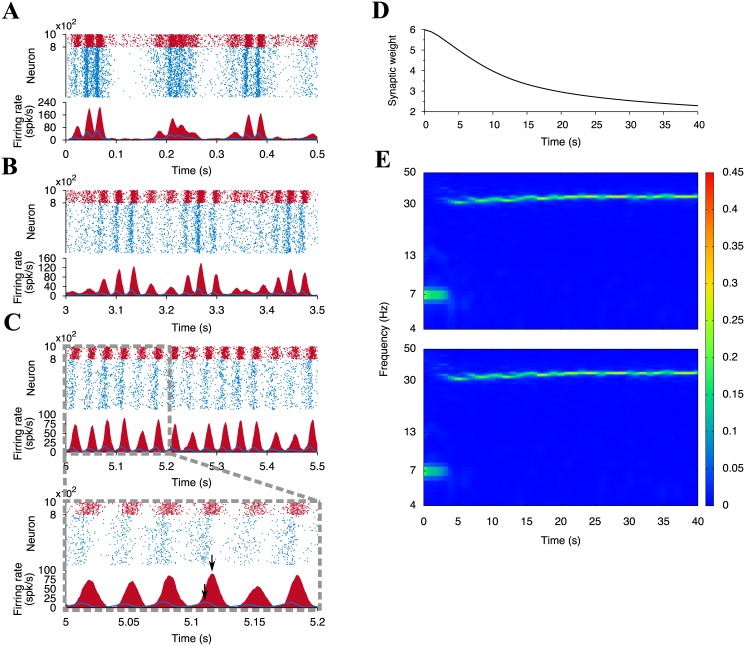
Neuronal activity and the mean synaptic weight in the network for 10 spk/s-external input. Rastergrams and the average firing rates of all excitatory (light blue) and all inhibitory (dark red) neurons at (A) *t* = 0 to 0.5 s, (B) 3 to 3.5 s, and (C) 5 to 5.5 s. The lower panel of (C) is the enlargement of the selected area 5–5.2 s of the upper panel. Two black arrows represent the peaks of the firing rates of excitatory and inhibitory neurons. The average firing rates are estimated by Eq (11). (D) Time course of the mean synaptic weight of the plastic synapses. (E) Power spectra of the firing rates of excitatory (upper) and inhibitory (lower) neurons. The colors indicate the normalized power intensity.

To show changes of the network activity during the learning process, we pick up the activity for 0–0.5 s, 3–3.5 s, and 5–5.5 s (Fig 6A–6C). The neural network exhibits oscillatory behaviors with frequency variations over time. At the beginning of the learning process, the network exhibits slow oscillation (Fig 6A), which speed up over time (Fig 6B and 6C). Additionally, the mean excitatory synaptic weight gradually decreases due to STDP and normalizes at around 2 (Fig 6D).

To gain further understanding, we conduct a spectrum analysis on these oscillations (Fig 6E). We notice that the time course of the power spectrum of the excitatory population looks very similar to that of the inhibitory population. This is explained as follows: By receiving the external inputs, excitatory and inhibitory neurons begin to fire and increase their firing rates. Triggered by the increase of the firing rate of the excitatory neurons, the inhibitory neurons are strongly activated. Indeed, the local maxima between the oscillations of excitatory and inhibitory neurons exhibit a small gap (Black arrows in Fig 6C bottom panel). Excessive firing of inhibitory neurons inactivates excitatory neurons by large amounts of negative feedback. This inhibition also leads to the silencing of the inhibitory neurons because of diminished excitatory inputs. The excitatory and the inhibitory neurons, however, are excited by the external inputs and begin to fire again. This cycle is repeated in the neural network, and therefore, this cycle results in the stable oscillation. This looks like the phenomenon known as the pyramidal-interneuronal gamma [49–52].

**Fig 7:**
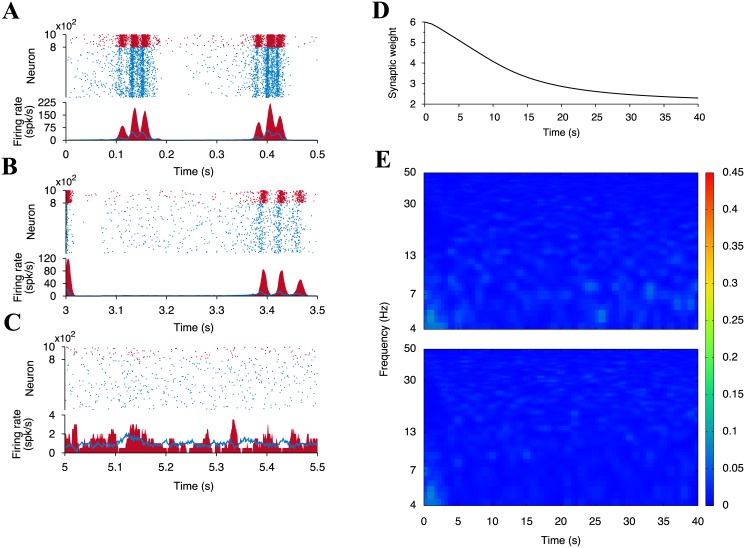
Same as Fig 6 but when the external input rate is 1 spk/s.

For comparison, we also observe the network behavior for the 1 spk/s-external input (Fig 7), where neural competition did not emerge (See also Fig 1D). Analogously to the 10 spk/s case, the network exhibits the slow oscillation at the early stages of learning (Fig 7A and 7B). As learning progress, the oscillation vanishes and the neuronal firing rate diminishes (Fig 7C). As seen in Fig 7E, no apparent peaks exist in the power spectra. Even though the settings are different than in the simulation using 10 spk/s-external input, the mean synaptic weight declines and normalizes at same point as in the case of 10 spk/s-external input (Fig 7D). A case comparison suggest that the neural oscillation supports enhancing synaptic potentiation more on the winner neurons than on the loser neurons.

### Mechanisms of neural competition

**Fig 8:**
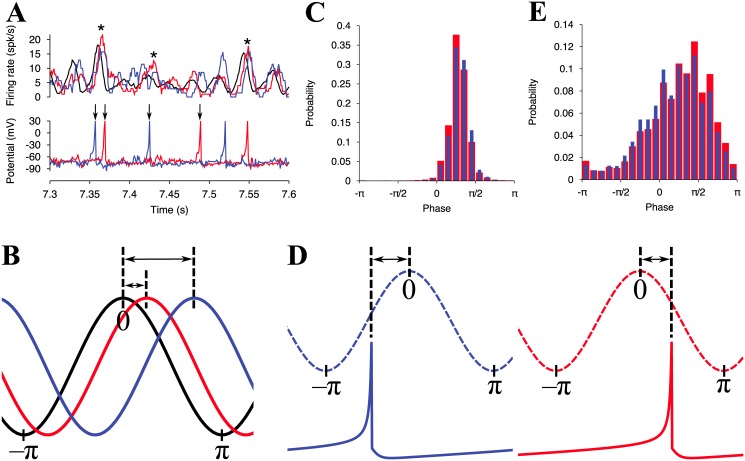
Mechanisms of emergence of winner and loser neurons in the neural network. (A) The lower panel is the time courses of the membrane potential of (red) a winner and (blue) a loser neuron. The upper panel is the average firing rates of (black) all excitatory neurons, (red) presynaptic terminals of the winner neuron, and (blue) presynaptic terminals of the loser neuron. These average firing rages are estimated by Eq (11). For the black line, timings of somatic firings are used. The red and the blue line are estimated with firing timings of presynaptic terminals, at which delay lengths are added to somatic firing timings of presynaptic neurons of the winner and the loser neuron. The winner and the loser neuron are randomly picked up from the identified groups at *t* = 3,600 s. (B) Schematic of a phase difference of the presynaptic oscillations from the global oscillation. The black, red and blue lines represent the mean firing rate of all excitatory neurons, the presynaptic terminals of the winner neuron, and the presynaptic terminals of the loser neuron, respectively. (C) Phase distributions of the local maxima in presynaptic terminal firing rate for the winner (red) and the loser (blue) neurons. The local maxima for the presynaptic firing rates are characterized by estimated phases relative to phases of a global firing rate of the excitatory neurons with Eq (10). The data for 45 s (from 5 s to 50 s) is used for the estimation. Results are however not different when using data after *t* = 50 s. Watson’s *U*^2^-test is used to test for significant differences in the distribution pattern between the winner and the loser neurons. This is the non-parametric test for phase data used to indicate any significant differences in the mean value or the variance (*P* < 0.001). (D) Schematic of the phase difference between the mean firing rate of presynaptic terminals and a postsynaptic firing in a winner neuron (left) and a loser neuron (right). The dashed and the solid lines represent the mean firing rate of presynaptic terminals and the postsynaptic potential, respectively. (E) Same as (C), but the local maxima of the presynaptic firing rates are replaced by the spikes of the winner (red) and the loser (blue) neuron firing rates (*P* < 0.001).

To understand the competition mechanisms, we observed presynaptic and postsynaptic activities of a winner and a loser neuron randomly selected from individual populations (Fig 8A).

The mean firing rates of the presynaptic terminals of the sampled winner and loser neurons oscillate in a similar manner as the firing rates of the excitatory neurons. However, the oscillation of the presynaptic terminals is slightly delayed from the oscillation of the excitatory neurons because of the axonal conduction delays (Fig 8A asterisks).

To quantify the delays, we evaluated gaps in the phases of the presynaptic oscillations from the excitatory oscillation as illustrated in Fig 8B. The mean probabilities of the phase gaps are plotted in Fig 8C. For both types of neurons, the positive gaps are collected, indicating that the presynaptic oscillations are delayed by the excitatory oscillation. The probability of phases gaps [0, *π*/4] in the winner neurons (red bars) is always higher than in the losers (blue bars). In contrast, the probabilities of the loser neurons exceeded those of the winner neurons in the larger phase range. This result is in good agreement with the previous result (Fig 5A) because the winner neurons have a higher ratio of smaller conduction delays. The distributions of the phase lags of the winners and the losers are significantly different (*P* < 0.001, Watson’s *U*^2^-test). For the details of Watson’s *U*^2^-test, see Materials and Methods.

To understand the potentiation and the depression processes in the neural oscillation, it is also necessary to observe the behaviors of the winner and the loser neurons. An example of the time trace of the membrane potentials of the sampled winner and the sampled loser neuron is depicted in Fig 8A (Lower panel). These neurons are the postsynaptic neurons of the synapses shown in Fig 8A (Upper panel). The winner neuron tends to fire just after the local maxima of its presynaptic firing rate (Black arrows for the red line in Fig 8A), while the loser neuron seems to emit spikes before the peaks of its presynaptic firing rate (Black arrows for the blue line in Fig 8A) as depicted in Fig 8D. Here, we characterized spikes of the winner and the loser neurons by using the phases of the presynaptic oscillations. The probability distributions of the phases are shown in Fig 8E. In both distributions, the largest peaks locate around *π*/2. In the range of [− *π*, 0], the probabilities of the loser neurons always exceed those of the winner neurons. The positions of the winners and the losers are reversed in [0, *π*/2]. This fact indicates that synapses on winner neurons are more potentiated and less depressed than those on loser neurons. The mean synaptic potentiation (or depression) between on winner neurons and on loser neurons is, on average, significantly different because there is significant difference in their probability distributions of phases (*P* < 0.001, Watson’s *U*^2^-test). Strong synapses are then, developed more on winner neurons than loser neurons.

### Influence of neural network parameters on neural competition

In the previous sections, we have showed that the cooperation of spontaneously induced oscillatory behaviors, axonal conduction delays, and learning interacted with each other and resulted in the neuronal competition. The behaviors of neurons and synapses must however be influenced by certain parameters in the network, which in turn affect neural competition. As such, we investigated the influence of various parameters on the neural competition.

The first two parameters are *α* determining the balance between LTD and LTP, and the inhibitory synaptic weight because they have strong impacts on the synaptic distributions [15, 16, 47, 49–52]. To evaluate organized neural network structures in these two parameter spaces, we used the instrength- and the outstrength-correlation coefficient. As shown in the results in Fig 3, when neural competition occurs, these coefficients become negative.

**Fig 9:**
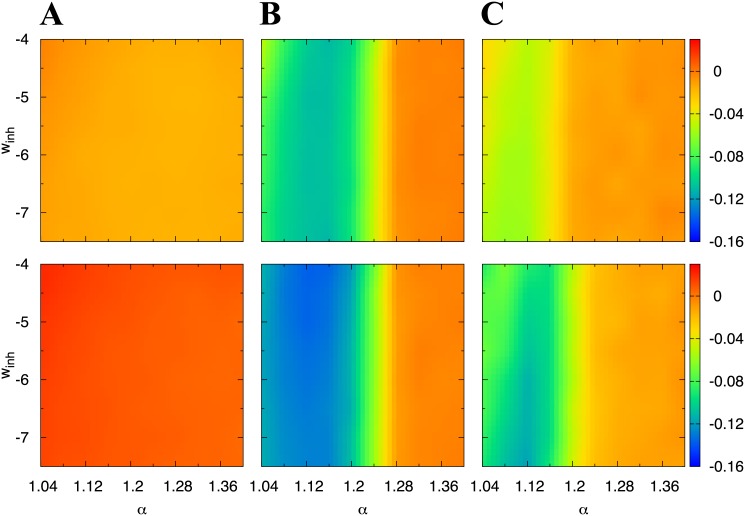
Influence of the balance between LTD and LTP and inhibition level on the organized networks. The firing rates of the external inputs are (A) 1 spk/s, (B) 10 spk/s, and (C) 40 spk/s. Colors correspond to the values of the instrength- (upper) and the outstrength- (lower) correlation coefficient. We plotted the instrength- and the outstrength-correlation coefficients of the neural network at *t* = 3,600 s for all the parameters.

As expected, these parameters drastically changed the network connectivity (Fig 9).

The network only established neural competition at 10 and 40 spk/s. In addition, the competition is observed for *α* ≤ 1.2. The parameter is in the suitable range of the experimentally observed STDP window function [12, 53]. The influence of the inhibition level on the competition is not observed in our result.

**Fig 10:**
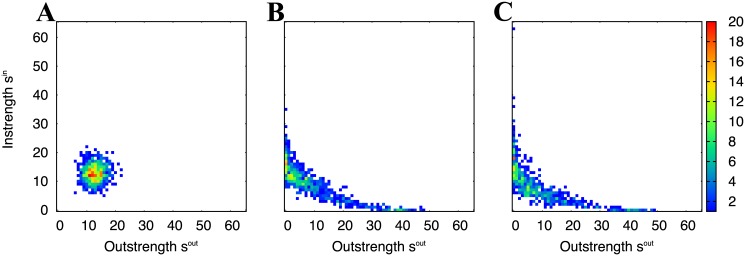
Influence of the upper bound of synaptic weights on the organized networks. The upper bound of synaptic weights is (A) *w*_*max*_ = 7, (B) *w*_*max*_ = 15, and (C) *w*_*max*_ = 20. The other parameters are the same as those in Fig 1B. The instrength- and outstrength-correlation coefficients are (A) 0.002 and −0.006, (B) 0.13 and 0.05, and (C) 0.3 and 0.2, respectively. All the results are obtained from the networks at *t* = 3,600 s.

The next parameter is the upper bound of plastic synapses. Any other parameters are the same as Fig 1B. In spite of the greater or the smaller maximum synaptic weight, neural competition does not emerge (*r*^in^ = 0.002 and *r*^out^ = 0.006 in Fig 10A, *r*^in^ = 0.13 and *r*^out^ = 0.05 in Fig 10B, *r*^in^ = 0.3 and *r*^out^ = 0.2 in Fig 10C). It can be considered that spike effects dramatically affects neural competition [54, 55].

### Influence of spike interactions in STDP on neural competition

In addition to certain parameters, STDP learning similarly influences neural competition in the network. In this section, we analyzed the influence of the spike interactions of STDP on neural competition. S2 File shows the JSDMs, when we simulated the neural network with the all-to-all interactions of STDP. All parameters of the network are the same as Fig 1. The neural competition is quantitatively and qualitatively identical to those in the case of the nearest-neighbor interactions. These results are expected because the all-to-all spike interactions only increase the frequency of the synaptic potentiation and depression and do not affect the competition mechanisms shown in the previous sections. Hence, the spike interactions in STDP are not a key factor for neural competition.

## Discussion

In this paper, we showed that excitable neurons in a recurrent network spontaneously competed and organized into two assemblies: winners and losers. Our analyses revealed that spontaneously induced neural oscillation, axonal conduction delays, and learning cooperated to establish neural competition.

In our numerical simulations, on average, STDP decreased synaptic weights in the network. At the same time, a certain level of neural activation spontaneously induced neural oscillation due to the existence of inhibitory neurons in the network. Around the local maxima of the oscillation, instantaneous firing rates of neurons in the network were high. If a postsynaptic neuron received inputs through many small delay connections, the inputs from the other neurons immediately arrived at the neuron. The spikes arrived before the next local oscillation maximum. Then, the presynaptic inputs could effectively induce firings of the postsynaptic neuron. This leads to potentiation of many synapses. Neurons that had high ratio of small axonal conduction delays were able to be winners. In contrast, the spike arrival at presynaptic terminals is delayed in neurons with many large delay connections. Therefore, many synapses failed to be potentiated. In addition, the LTD window is dominant in STDP, which already depressed the synapses in the network. For these reasons, the neurons with many large delay connections became the losers.

Because a specific memory is thought to be stored in a subnetwork consisting of a small population of neural circuits [1, 2], the neural competition shown in this paper is an important property for memory formation [3–6]. The competition mechanism in this paper is applicable to real neural circuits because all key factors of competition are observable in neural circuits. Because the length of a axonal conduction delay is proportional to the distance between a pair of neurons, our result indicates that synaptic connections of closer neurons were strengthened by neural oscillations. In other words, the neurons organized locally dense but globally sparse neural circuits. This might be related to the distance-dependent high-order correlations of neuronal activities [7, 8]. It is implied that highly nonrandom local connectivity is organized by the distance-dependent high-order correlations [36, 37].

In our analyses, the neural competition is accomplished when certain parameters of numerical simulations were set within physiologically reasonable limits. For example, the firing rate of external inputs, the slight dominance of the depression in the STDP window, and the upper bound of plastic synapses. The parameters tested in this paper might be unique in different areas of the brain [9, 56]. As shown in this paper, it is suggested that local circuits organize very differently in different brain areas.

Iglesias et al. conducted similar simulations and analyses using the leaky-integrate and fire units and the additive STDP, in which spike interaction is the nearest neighbor, without axonal conduction delays [23]. They extended their simulations and analyses by introducing neuron death with larger size of neural networks [57, 58]. In their simulations of Ref. [23], they assumed the application of inputs to a fraction of the population, that shaped a bar column, that dynamically moved, in 2D lattice. In Fig 4 of Ref. [23], Eqs (5) and (6) were used in their analyses. In the result, the neurons exhibited clear competition in the excitatory neural population. The indgrees in two neural groups were much different but their outdegrees were the same level. However, our analyses with the degrees show a reverse trend. The main difference of our simulation settings from Ref. [23] is the way of the application of inputs and the existence of conduction delays. In our result, we do not observe large difference of indegrees between the two groups (S2 Fig). Rather, there is the gap of outdegrees between them. This difference might come from the effect of the way of the external stimulation and the axonal conduction delays, especially the latter one is important for the neural competition shown in the current study because of the competition mechanisms (See also the section of Mechanisms of neural competition). Then, it is considered that the competition in the STDP neural networks in the current study originates in the different mechanisms from ones of Ref. [23]. So, it is suggested that there are some possible mechanisms of the neural competition. Accordingly, the neural competition in STDP recurrent networks should be further analyzed.

## Supporting Information

S1 File Influence of initial synaptic distributions on the organization of networks. Initial excitatory synaptic weights have a uniform distribution in the range of (Figure A) [5, 7], (Figure B) [4, 8], and (Figure C) [3, 9]. The panels show JSDMs. The instrength- and outstrength-correlation coefficients for each case are (Figure A) − 0.09 and − 0.11, (Figure B) − 0.09 and − 0.11, and (Figure C) − 0.09 and − 0.11, respectively. The external input rate is fixed to 10 spk/s. All the results are obtained from the neural networks at *t* = 3,600 s.
(EPS)

S2 File Influence of spike interactions of STDP on the organized networks. All spike pairs contribute to synaptic modifications (all-to-all interaction) [15–18]. The panels show the JSDMs after the STDP learning (*t* = 3,600 s) with (Figure A) 1 spk/s, (Figure B) 10 spk/s, and (Figure C) 40 spk/s external inputs. The instrength and outstrength correlation coefficients are (Figure A) − 0.01 and 0.008, (Figure B) − 0.09 and − 0.1, and (Figure C) − 0.03 and − 0.03, respectively.
(EPS)

S1 Fig The stability of the synaptic weight distributions for 1 spk/s (gray), 10 spk/s (red) and 40 spk/s (blue) external input rate. Left panels are enlargements of right panels for 1–50 s. All the parameters of the network and STDP implementation are the same as in Fig 1. The stability is quantified with the Jensen-Shannon divergence between the synaptic distributions at *t* and *t* + 1 s.
(EPS)

S2 Fig Neurons are not apparently competitive from a viewpoint of their indegrees. Joint degree distribution matrices (JDDMs) of 10 spk/s. For JDDMs, Eqs (5) and (6) were used instead of Eqs (7) and (8).
(EPS)
